# Analysis of T and B Cell Epitopes to Predict the Risk of *de novo* Donor-Specific Antibody (DSA) Production After Kidney Transplantation: A Two-Center Retrospective Cohort Study

**DOI:** 10.3389/fimmu.2020.02000

**Published:** 2020-08-27

**Authors:** Shintaro Sakamoto, Kenta Iwasaki, Toshihide Tomosugi, Matthias Niemann, Eric Spierings, Yuko Miwa, Kosei Horimi, Asami Takeda, Norihiko Goto, Shunji Narumi, Yoshihiko Watarai, Takaaki Kobayashi

**Affiliations:** ^1^Department of Renal Transplant Surgery, Aichi Medical University School of Medicine, Nagakute, Japan; ^2^Department of Histocompatibility Laboratory, Nagoya Daini Red Cross Hospital, Nagoya, Japan; ^3^Department of Kidney Diseases and Transplant Immunology, Aichi Medical University School of Medicine, Nagakute, Japan; ^4^Department of Transplant Surgery, Nagoya Daini Red Cross Hospital, Nagoya, Japan; ^5^PIRCHE AG, Berlin, Germany; ^6^Laboratory of Translational Immunology, UMC Utrecht, Utrecht, Netherlands; ^7^Department of Nephrology, Nagoya Daini Red Cross Hospital, Nagoya, Japan; ^8^Department of Transplant Internal Medicine, Nagoya Daini Red Cross Hospital, Nagoya, Japan

**Keywords:** kidney transplantation, eplet mismatch, PIRCHE-II, donor specific antibody, epitope analysis

## Abstract

Risk prediction of *de novo* donor specific antibody (DSA) would be very important for long term graft outcome after organ transplantation. The purpose of this study was to elucidate the association of eplet mismatches and predicted indirectly recognizable HLA epitopes (PIRCHE) scores with *de novo* DSA production. Our retrospective cohort study enrolled 691 living donor kidney transplantations. HLA-A, B, DRB and DQB eplet mismatches and PIRCHE scores (4 digit of HLA-A, B, DR, and DQ) were determined by HLA matchmaker (ver 2.1) and PIRCHE-II Matching Service, respectively. Weak correlation between eplet mismatches and PIRCHE scores was identified, although both measurements were associated with classical HLA mismatches. Class II (DRB+DQB) eplet mismatches were significantly correlated with the incidence of *de novo* class II (DR/DQ) DSA production [8/235 (3.4%) in eplet mismatch ≤ 13 vs. 92/456 (20.2%) in eplet mismatch ≥ 14, *p* < 0.001]. PIRCHE scores were also significantly correlated with *de novo* class II DSA production [26/318 (8.2%) in PIRCHE ≤ 175 vs. 74/373 (19.8%) in PIRCHE ≥ 176, *p* < 0.001]. Patients with low levels of both class II eplet mismatches and PIRCHE scores developed *de novo* class II DSA only in 4/179 (2.2%). Analysis of T cell and B cell epitopes can provide a beneficial information on the design of individualized immunosuppression regimens for prevention of *de novo* DSA production after kidney transplantation.

## Introduction

Chronic antibody-mediated rejection (ABMR) caused by *de novo* donor specific antibody (DSA) is a major cause of graft failure in solid organ transplantation (1). Randomized clinical trials have been undertaken in order to explore the efficacies of various treatments for ABMR (2). Although intravenous immunoglobulin (IVIG) and plasmapheresis have been advocated as standard of care, particularly in cases of acute ABMR, there are no effective treatments for chronic ABMR that would prevent the gradual deterioration of graft function (3). A means to prevent chronic ABMR is likely to be far superior than any available cure (4). While not all DSAs promote ABMR (5–8), the development of *de novo* DSAs remains among the most definitive of the known risk factors that promote this adverse event. Therefore, risk prediction of *de novo* DSA would be important for long term graft outcome.

Recently, a rigorous analysis of B cell epitopes was conducted in order to assess the immunogenicity of HLA mismatch in greater detail (9). The HLAMatchmaker algorithm was developed based on the concept of the HLA molecule as a linear sequence of amino acid triplets and via evaluation of the eplets, which are the small three-dimensional structure of amino acid residues that are the essential components of immunogenicity. Results from HLA epitope matching based on this concept have been reported to be superior to those obtained from more conventional HLA matching modalities. This new methodology provides greater insight into the risk of developing *de novo* DSAs as well as the possibility of reorganizing the organ allocation system (10). Many research groups have explored this issue, and reported that the degree of epitope mismatches recognized by B cell receptors as defined by an eplet, amino acid sequence and electrostatic mismatch would have a significant correlation with DSA production, ABMR and graft outcome in organ transplantation (11–19).

In parallel with B cell epitopes, attention has also been focused on T cell epitopes, specifically, those associated with donor-derived HLA molecules presented by HLA class II on recipient antigen presenting cells (20). T cell epitopes are recognized by the T cell receptor of CD4+ T cells at the **first** step toward DSA production via T-dependent B cell activation (Supplementary Figure 1). The number of potential T cell epitopes has been correctly assessed by the PIRCHE (Predicted indirectly recognizable HLA epitopes)-II algorithm (21, 22).

The purpose of this study was to examine the association of the eplet mismatch level and PIRCHE scores with *de novo* DSA production after kidney transplantation. Our goal was to elucidate the clinical significance of both T cell and B cell epitope prediction as a risk factor for *de novo* DSA production.

## Materials and Methods

### Study Design and Subjects

We conducted a retrospective cohort study of adult patients (*n* = 793) who underwent living donor kidney transplantation at Aichi Medical University or the Nagoya Daini Red Cross Hospital between 2008 and 2015. We excluded recipients with pre-existing DSAs (*n* = 66) and those who were lost to follow-up within 1 year due to death (*n* = 3), graft failure (*n* = 5) or transfer of care to a remote hospital (*n* = 28). The remaining 691 patients were enrolled in the retrospective cohort study. The final date for the analysis of graft survival was April 30, 2019; the mean follow-up period after transplantation was 78.7 ± 27.7 months.

### HLA Typing, Eplet Mismatch and PIRCHE Score

HLA (-A, -B, -DRB1, -DQA1, and -DQB1) typing of donors and recipients was performed by xMAP® Technology of Luminex Corp. using PCR-sequence specific oligonucleotide (SSO) probes (Wakunaga Pharmaceutical Co. Ltd., Hiroshima, Japan or One Lambda, Canoga Park, CA, USA) at high resolution. Some low-resolution typing or missing data on HLA-A, B and DQA1 were extrapolated to second field HLA typing using the HLA-haplotype frequencies in Japanese population (23). Eplet mismatches and PIRCHE scores were determined using HLA types at four-digit levels. Eplet mismatch levels for HLA class I (A, B) and class II (DRB1, 3, 4, 5, and DQB1) were determined by HLAMatchmaker software v2.1. PIRCHE scores were calculated as a sum of mismatched HLA-A, HLA-B, HLA-DRB1, 3, 4, 5, HLA-DQA1, and HLA-DQB1-derived peptide counts presented with respect to the recipients' HLA-DRB1, 3, 4, 5, HLA-DQA1, and HLA-DQB1 using PIRCHE-II algorithm via the matching service.

### Anti-HLA Antibody Detection and Identification of DSAs

For all recipients, anti-HLA antibodies were analyzed before transplantation and monitored annually after transplantation. Serum samples collected from 2009 to 2019 were examined for IgG antibodies against HLA class I or II using methodologies including Flow PRA, LABScreen Mixed and LABScreen PRA (One Lambda). Any positive evaluations were re-screened and the DSA was identified using LABScreen Single Antigen and Supplement (One Lambda). Mean fluorescence intensity (MFI) values above 1,000 for DSAs against HLA-A, -B, -DR, and -DQ at the 4-digit level were scored as positive.

### Immunosuppressive Agents

All the patients received 500 mg of intravenous (IV) methylprednisolone prior to graft reperfusion and 20 mg of IV basiliximab as induction therapy on days 0 and 4. Maintenance immunosuppressive therapy consisted of a calcineurin inhibitor (cyclosporine or tacrolimus), steroid (prednisolone), and antimetabolites (mycophenolate mofetil or mizoribine) or an mTOR inhibitor (everolimus). Dosages of all oral immunosuppressive medications except for prednisolone were strictly adjusted according to pharmacokinetics, including area under the curve (AUC) or trough levels (24). Recipients of ABO-incompatible transplants were additionally pre-treated with mycophenolate mofetil from day−14 as well as double-filtration plasmapheresis and either splenectomy, rituximab (200 mg/body) on day−14 and/or day−1 or neither (due to low anti-A/B antibody titers).

### Statistical Analysis

Statistical analyses were performed using JMP software v13.2. Nominal variables were examined using Fisher's exact test or chi-square test. Continuous variables were presented as mean ± SD and analyzed by Student's *t*-test or Mann-Whitney *U*-test. Spearman's rank correlation and simple linear regression analysis were conducted for quantifying the association. Receiver operating characteristic (ROC) curve analyses were performed to obtain the best predictive value of eplet mismatches and PIRCHE scores. Logistic regression model for univariate and multivariate analysis was used to assess the valuables associated with DSA production. DSA-free graft survival was defined as the time between kidney transplantation and the date of final follow-up without DSA detection. DSA-free survival rates were estimated using Kaplan-Meier survival curves and Wilcoxon tests. Cox proportional hazards regression model for univariate and multivariate analysis was used to find variables that impacted DSA-free survival. *P* < 0.05 were considered to be statistically significant.

## Results

### *De novo* DSA Production

*De novo* DSAs were detected in 114 (16.5%) of the 691 recipients enrolled in this study, including antibodies targeting HLA-class I (*n* = 14), class I + DR (*n* = 1), class I+ DQ (*n* = 2), DR (*n* = 19), DQ (*n* = 69) and DR + DQ (*n* = 9). DSAs detected were predominantly those directed against class II (*n* = 100), most notably HLA-DQ followed by HLA-DR. The incidence of HLA class I DSA was comparatively low; most of the HLA class I DSAs presented with low MFIs that fluctuated around cutoff level. MFI levels of class I DSA and class II DSA were 2,481 +/- 2,073, and 11,404 +/- 8,389, respectively. Chronic ABMR was reported to be mainly associated with HLA class II DSA (5, 6, 11, 12, 19, 25, 26). For these reasons, only class II (DR and/or DQ) DSAs were considered in the risk assessment.

### Patient Data

Donor gender, classical HLA-A and B mismatches, eplet mismatches of HLA-A and B, and DRB showed statistically significant difference between *de novo* HLA-class I DSA-positive (*n* = 17) and negative (*n* = 674) patients, although positive number might be too small for precise analysis (Table 1A). There were no significant differences with respect to observation period, recipient/donor age, gender, relationship, use of basic immunosuppressive agents, use of desensitization therapy, or incidence of CMV infection between the *de novo* DR/DQ DSA-positive (*n* = 100) and negative patients (*n* = 591; Table 1B). Furthermore, no significant differences in levels of classical HLA mismatch were detected. A history of acute T cell-mediated rejection and blood group ABO compatibility were both identified as significant risk factors.

**TABLE 1A:** Patient Characteristics by *de novo* class I DSA status.

**Factors**	***De novo* A/B**	***De novo* A/B**	***P-*value**
	**DSA (+) (*n* = 17)**	**DSA (−) (*n* = 674)**	
Observation period	85.7 +/– 28.4	78.5 +/– 27.6	
Recipient age	46.3 +/– 15.4	16.3 +/– 15.9	
Recipient M/F	14/3	437/237	
Donor age	56.9 +/– 11.1	58.0 +/– 10.1	
Donor M/F	2/15	240/434	0.0422
Parent/sibling/spouse/others	7/1/9/0	282/60/305/27	
CSA/TAC	12/5	340/434	
MMF/MZR/EVR	13/4/0	565/34/75	
RIT/SPX/NONE	4/2/11	170/8/496	
HLA-A+B MM	2.6 +/– 0.8	2.1 +/– 1.0	0.0446
HLA-DRB1 MM	1.2+/– 0.8	1.2 +/– 0.6	
HLA-DQB1 MM	1.2 +/– 0.7	1.1 +/– 0.6	
HLA-DRB1+DQB1 MM	2.4 +/– 1.5	2.4 +/– 1.2	
HLA-A+B+DRB1+DQB1 MM	5.1 +/– 2.0	4.5 +/– 2.0	
ABO-I/ABO-Id/C	6/11	231/443	
Eplet MM (A+B)	15.7 +/– 5.2	11.1 +/– 6.4	0.0033
Eplet MM (DRB)	7.1 +/– 7.0	11.4 +/– 8.8	0.0426
Eplet MM (DQB)	9.8 +/– 7.2	8.3 +/– 6.5	
Eplet MM (DRB+DQB)	16.9 +/– 12.6	19.7 +/– 13.3	
PIRCHE score	211.9 +/– 136.7	214.0 +/– 139.7	
Acute TCMR	0 (0%)	56 (8.3%)	
CMV infection	2 (26.0%)	196 (29.1%)	

**TABLE 1B:** Patient Characteristics by *de novo* class II DSA status.

**Factors**	***De novo* DR/DQ**	***De novo* DR/DQ**	***P-*value**
	**DSA (+) (*n* = 100)**	**DSA (−) (*n* = 591)**	
Observation period	80.8 +/– 28.7	78.3 +/– 27.5	
Recipient age	44.7 +/– 17.4	46.6 +/– 15.6	
Recipient M/F	73/27	378/213	0.0886
Donor age	56.9 +/– 11.1	58.0 +/– 10.1	
Donor M/F	29/71	213/378	
Parent/sibling/spouse/others	45/8/38/9	244/53/276/18	
CSA/TAC	60/40	292/299	0.0522
MMF/MZR/EVR	77/8/15	501/30/60	
RIT/SPX/NONE	20/2/78	154/8/429	
HLA-A+B MM	2.2 +/– 0.9	2.2 +/– 1.0	
HLA-DRB1 MM	1.3 +/– 0.5	1.2 +/– 0.6	
HLA-DQB1 MM	1.3 +/– 0.5	1.1 +/– 0.7	
HLA-DRB1+DQB1 MM	2.6 +/– 0.9	2.3 +/– 1.3	
HLA-A+B+DRB1+DQB1 MM	4.8 +/– 1.6	4.5 +/– 2.0	
ABO-I/ABO-Id/C	24/76	213/378	0.0223
Eplet MM (A+B)	11.0 +/– 6.8	11.2 +/– 6.4	
Eplet MM (DRB)	15.0 +/– 7.9	10.7 +/– 8.8	<0.0001
Eplet MM (DQB)	11.0 +/– 5.7	7.9 +/– 6.5	<0.0001
Eplet MM (DRB+DQB)	25.9 +/– 10.9	18.6 +/– 13.3	<0.0001
PIRCHE score	265.5 +/– 139.1	205.3 +/– 137.8	<0.0001
Acute TCMR	20 (20.0%)	36 (6.1%)	<0.0001
CMV infection	26 (26.0%)	172 (29.1%)	

*DSA, donor specific antibody; CSA, cyclosporine; TAC, tacrolimus; MMF, mycophenolate mofetil; MZR, mizoribine; EVR, everolimus; RIT, rituximab; SPX, splenectomy; MM, mismatch; ABO-I, ABO-incompatible transplantation; ABO-Id/C, ABO-identical/compatible transplantation; TCMR, T cell mediated rejection*.

### Association Between Eplet Mismatch and PIRCHE Score

**Figure 1:**
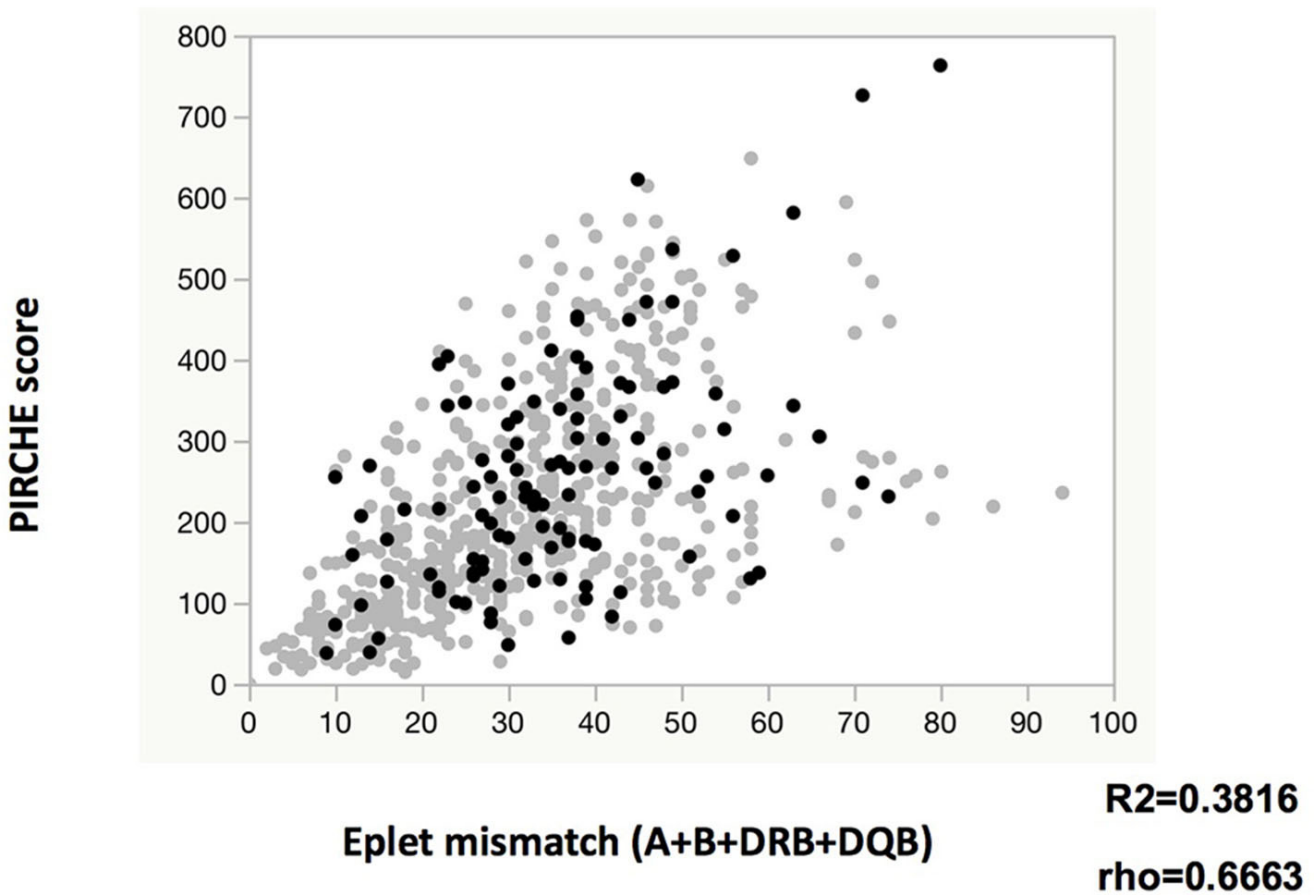
Relationship between eplet mismatch and PIRCHE score. Gray circles and black circles indicate *de novo* DR/DQ DSA negative and positive, respectively.

The eplet mismatches of class I (A+B) and class II (DRB, DQB, or both) were significantly higher among the *de novo* class I and class II DSA-positive patients than among those who were *de novo* DSA-negative, respectively (Tables 1A,B). PIRCHE scores were significantly higher in the *de novo* class II DSA-positive patients than in those were DSA-negative. Both eplet mismatch levels and PIRCHE scores were associated with classical HLA mismatches (Supplementary Figures 2A–C), there were weak positive correlations between eplet mismatches (A, B, DRB, and DQB) and PIRCHE scores (Figure 1). Compared to DSA-negative patients, DSA-positive patients tended to have a higher degree of eplet mismatches and higher PIRCHE scores. However, DSA-positive patients showed positive correlation between eplet mismatches and PIRCHE score to a lesser extent (DSA-positive; *R*^2^ = 0.2340, rho = 0.4621 vs. DSA-negative; *R*^2^ = 0.3940, rho = 0.6968).

### *De novo* DR/DQ DSA Production Associated With Eplet Mismatch and PIRCHE Score

ROC curve revealed that the number of eplet mismatches = 14 and PIRCHE score = 176 provide the best predictive values (Supplementary Figure 2). Eplet mismatches were significantly associated with the incidence of *de novo* DSAs, observed in 8/235 (3.4%) of those with eplet mismatches ≤ 13 vs. 92/456 (20.2%) of those with eplet mismatches ≥ 14 (14–67 vs. 0–13: OR 7.172, 95% CI 3.418–15.050, *p* < 0.0001; Table 2A). PIRCHE scores were also significantly associated with *de novo* DSA production, identified in 26/318 (8.2%) of those with PIRCHE scores ≤ 175 and 74/373 (19.8%) among those with PIRCHE scores ≥ 176 (176–763 vs. 0–175: OR 2.780, 95% CI 1.728–4.470, *p* < 0.0001; Table 2B). Patients were divided into groups, including those with eplet mismatches of 0, 1–13, and 14–67, and PIRCHE scores of 0, 1–175, 176–763 as per the optimized predictive value based on ROC curve analysis. Only 4/99 (4.0%) of the patients with low levels of both eplet mismatches (1–13) and PIRCHE scores (1–175) produced *de novo* DSAs, whereas, *de novo* DSAs were detected in 70 (22.1%) of 317 patients with high levels of both parameters (Table 2C).

**TABLE 2:** Incidence of *de novo* DR/DQ DSA by (A) eplet mismatch and (B) PIRCHE score, (C) *de novo* DSA positive rate by both of eplet mismatch and PIRCHE score.

		***De novo*** **DR/DQ DSA**		
		**(+)**	**(–)**	**Positive rate**
**(A)**				
**Eplet MM (DRB+DQB)**	**0–13**	**8**	**227**	**3.4%**
	**14–67**	**92**	**364**	**20.2%**
				***P* < 0.0001**
**(B)**				
**PIRCHE score**	**0–175**	**26**	**292**	**8.2%**
	**176–763**	**74**	**299**	**19.8%**
				***P* < 0.0001**
**(C)**				
		**Eplet MM (DRB+DQB)**		
		**0**	**1–13**	**14–67**
PIRCHE score	0	0/28 (0%)	0/0 (0%)	0/0 (0%)
	1–175	0/52 (0%)	4/99 (4.0%)	22/139 (15.8%)
	176–763	0/2 (0%)	4/54 (7.4%)	70/317 (22.1%)

### DSA-Free Graft Survival Predicted by Eplet Mismatch and PIRCHE Score

**Figure 2:**
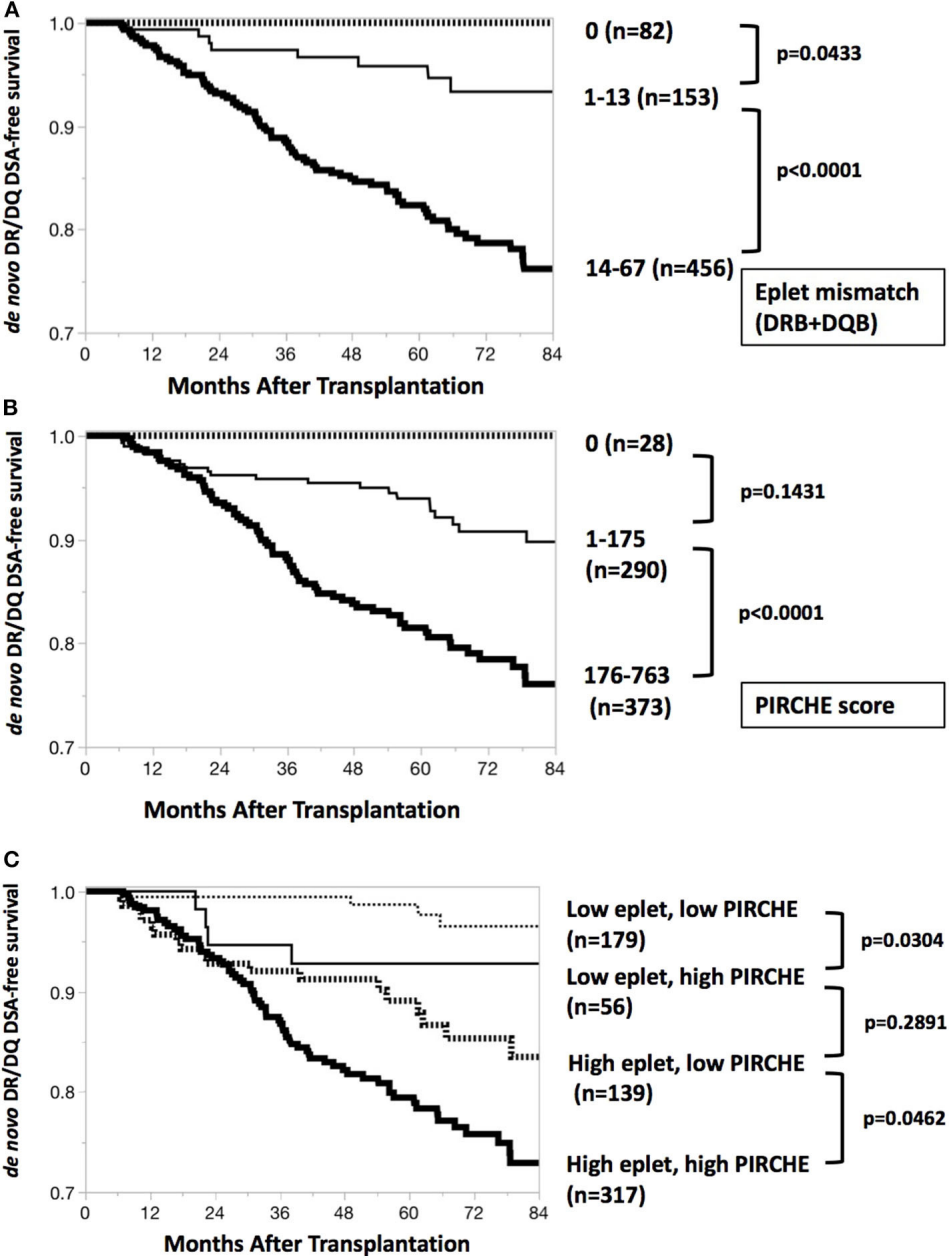
*De novo* DR/DQ DSA-free survival curves. **(A)** *De novo* DR/DQ DSA-free survival by eplet mismatch. Kaplan-Meier *de novo* DR/DQ-free survival curves and Wilcoxon test show significant difference (*p* < 0.0001) between epitope mismatches 1–13 and 14–67. Thick dotted line, thin black line and thick black line indicate DSA-free survival (months after transplantation) of groups with eplet mismatches 0, 1–13, and 14–67, respectively. **(B)** *De novo* DR/DQ DSA-free survival by PIRCHE score. Kaplan-Meier *de novo* DR/DQ-free survival curves and Wilcoxon test show significant difference (*p* < 0.0001) between PIRCHE scores 1–175 and 176–763. Thick dotted line, thin black line and thick black line indicate DSA-free survival (months after transplantation) of groups with PIRCHE scores 0, 1–175, and 176–763, respectively. **(C)** *De novo* DR/DQ DSA-free survival by both eplet mismatch and PIRCHE score. Subgroup analysis by Kaplan-Meier *de novo* DR/DQ-free survival curves and Wilcoxon test shows significant differences between low and high PIRCHE scores among patients with low eplet mismatches (*p* = 0.0304) and high eplet mismatches (*p* = 0.0462). Thin dotted line, thin black line, thick dotted line and thick black line indicate DSA-free survival (months after transplantation) of groups including low eplet + low PIRCHE, low eplet + high PIRCHE, high eplet + low PIRCHE, and high eplet + high PIRCHE, respectively. Low eplet mismatches = 0–13; high eplet mismatches = 14–67; low PIRCHE scores = 0–175; high PIRCHE scores = 176–763.

*De novo* DR/DQ DSA-free graft survivals by eplet mismatch and PIRCHE score were depicted in Figures 2A,B. Patients with eplet mismatches from 14 to 67 or PIRCHE scores from 176 to 763 responded with a significantly higher incidence of *de novo* DSAs. Subgroup comparisons revealed that higher PIRCHE scores were also associated with a significantly higher incidence of *de novo* DSAs than were low PIRCHE scores; this was the case among patients grouped in either the low or high eplet mismatch group (Figure 2C).

Multivariate analysis of a Cox hazard regression model revealed that ABO-compatibility, eplet mismatch, PIRCHE score and history of acute T cell mediated rejection were all significantly associated with *de novo* DSA production (Table 3).

**TABLE 3:** Risk factors associated with *de novo* DR/DQ DSA production.

**Cox proportional hazards regression model (DR, DQ)**				
**Variables**	**Univariate analysis**		**Multivariate analysis**	
	**HR (95% CI)**	***p*****-value**	**HR (95% CI)**	***p*****-value**
CSA vs. TAC	1.314 (0.883–1.977)	0.179		
ABO-I vs. ABO-Id/C	0.613 (0.379–0.954)	0.0295	0.465 (0.283–0.736)	0.0009
Eplet MM (DRB+DQB)	1.035 (1.021–1.049)^*^	<0.0001	1.026 (1.009–1.043)^*^	0.0028
PIRCHE score	1.026 (1.013–1.039)^**^	0.0001	1.016 (1.001–1.032)^**^	0.0347
Acute TCMR	3.052 (1.819–4.880)	<0.0001	3.309 (1.937–5.409)	<0.0001

* Hazard ratio for one unit increase is expressed.

** Hazard ratio for 10 unit increase is expressed.

*HR, Hazard ratio; CI, confidence interval; CSA, cyclosporine; TAC, tacrolimus; ABO-I, ABO-incompatible transplantation; ABO-Id/C, ABO-identical/compatible transplantation; MM, mismatch; TCMR, T cell mediated rejection*.

## Discussion

Early diagnosis for chronic ABMR may be essential, because most treatment would be ineffective once there is evidence of graft dysfunction including elevated levels of serum creatinine and overt proteinuria (6). Effective treatment under subclinical condition of ABMR has been recently reported (27). However, early detection of ABMR is somewhat difficult due to consensus guidelines that suggest that routine monitoring of DSA monitoring is overall not cost-effective (28, 29). Furthermore, even if annual monitoring for all transplant patients was implemented, this interval might be too prolonged for meaningful detection of DSAs in some patients. The issue on the frequency of HLA antibody monitoring remains to be clarified. At the same time, innovative methods including high throughput technology and bioinformatics are currently in use in an effort to identify a biomarker for early detection of ABMR (30–32). Preventive measures are currently considered to be more likely to provide superior patient care when compared to the impact of therapeutic or preemptive strategies.

Our finding that the correlation between eplet mismatches and PIRCHE scores was weaker in DSA-positive patients than in DSA-negative patients corresponds to the previous report (33), although eplet mismatches for class I DSA and PIRCHE scores for donor-derived HLA class I were analyzed at that time. Two approaches, eplet mismatches and PIRCHE scores, seemed to be complementary to each other for predicting the risk of DSA production.

We found that analyses of both B cell and T cell epitopes (i.e., eplet mismatches and PIRCHE scores, respectively), had positive predictive capabilities with respect to *de novo* DSA production. Recent studies reported strong associations of eplet mismatch and/or PIRCHE-II scores with *de novo* DSA production or graft outcomes (25, 26, 34, 35), whereas it was also reported that allelic and epitope mismatch analysis presented no additional value with respect to risk management (36). Differences in immunogenicity might be dependent on the nature of the eplet and the precise mismatch position (37, 38). The immunological impact of PIRCHE-II is determined by the interactions between T cell receptor and the donor-derived peptides presented by HLA class II (22). Complete development of these algorithms in order to take into account both T and B cell epitopes remains a substantial challenge.

Our study has several limitations. This retrospective study features a relatively small sample size and brief follow-up period. There was substantial heterogeneity with respect to the immunosuppressive protocols used; this was to some extent related to the multi-center nature of this study. Our eplet mismatch data (primarily associated with HLA-DRB1,3,4,5, and DQB1) were comparable to findings from previous reports (12, 16, 17, 19, 25, 26, 34), although class I DSAs were not considered in this analysis. We calculated PIRCHE score based on typing information of recipient HLA-DRB1,3,4,5, DQA1, and DQB1 as the presenting HLA class II molecules; the addition of DRB3,4,5, and DQA1/DQB1 explains why our PIRCHE scores were higher in range than those included in previous reports that were based on DRB1 alone (21, 34, 35), even though we did not consider HLA-C among the donor-derived peptides. Patients with preformed DSAs were excluded so that our study could focus on primary immune responses rather than memory responses associated with long-term graft outcome (39). The primary endpoint in this study was *de novo* DSA production, not chronic ABMR or graft failure. Further analysis that included these data could potentially provide more convincing evidence of clinical benefit of the analyses of T cell and B cell epitopes; this would require significantly longer follow-up times. Furthermore, we need to consider the fact that *de novo* DSA production does not necessarily result in ABMR; this incidence of ABMR may relate to the amount and specificity of the DSAs, their ability to bind complement as well as graft accommodation resulting from ABO-incompatibility (7, 8, 40, 41). It was recently reported that imputed HLA alleles could lead to false findings particularly in multi-ethnic non-Caucasian individuals (42). Although we cannot deny such a possibility, we expect that single ethnic subjects used in this study would reduce the risk of estimation error.

Despite the above-mentioned limitations, the currently available data have clearly revealed the potential value of epitope analysis. These modalities offer predictive factors that are more reliable with respect to *de novo* DSA production than conventional HLA matching. This enhanced reliability suggests that these methodologies are likely to provide important contributions toward the development of individualized immunosuppression strategies in the not too distant future.

In conclusion, our findings revealed significant associations of eplet mismatches and PIRCHE scores with the prevalence of *de novo* DSAs. Further analysis of T cell and B cell epitopes is likely to provide critical information for the development of individualized immunosuppression strategies for the prevention graft rejection. Computer-based algorithms that predict T cell and B cell epitopes are undergoing rapid development. Further study will be needed in order to draw definitive conclusions regarding the clinical value of these predictive algorithms with respect to ABMR after organ transplantation.

## Data Availability Statement

The raw data supporting the conclusions of this article will be made available by the authors, without undue reservation.

## Ethics Statement

The studies involving human participants were reviewed and approved by The Institutional Ethics Committees of Aichi Medical University Hospital and the Institutional Review Board of Nagoya Daini Red Cross Hospital. The patients/participants provided their written informed consent to participate in this study.

## Author Contributions

SS, KI, TT, and TK designed the research. SS and TK wrote the manuscript. SS, TT, KH, AT, NG, SN, YW, and TK performed the research. SS, TT, MN, ES, and TK participated in data analysis. ES and TK reviewed/edited the manuscript. All authors contributed to the article and approved the submitted version.

## Conflict of Interest

MN is an employee of PIRCHE AG. The UMC Utrecht has filed a patent application on the prediction of an alloimmune response against mismatched HLA. ES is listed as inventor on this patent. The remaining authors declare that the research was conducted in the absence of any commercial or financial relationships that could be construed as a potential conflict of interest.
