# Supplementary material for: Analysis of T and B Cell Epitopes to Predict the Risk of de novo Donor-Specific Antibody (DSA) Production After Kidney Transplantation: A Two-Center Retrospective Cohort Study
Source: Front Immunol. 2020 Aug 27;11:2000. doi: 10.3389/fimmu.2020.02000 (PMC7481442; doi:10.3389/fimmu.2020.02000)
Supplement: Supplementary file 1 [file Image_1.pdf]

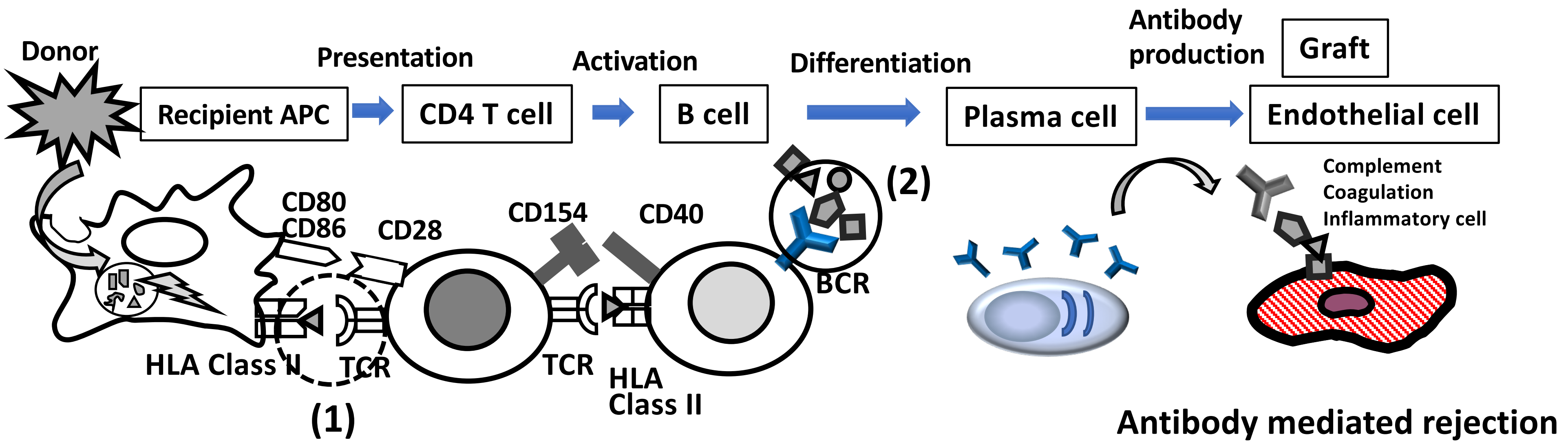

Supplementary Figure 1. T cell and B cell epitopes in the process of allorecognition and HLA antibody production

After presentation of donor-derived HLA peptides by HLA class II on recipient APC, CD4+ follicular helper T cells facilitate proliferation and differentiation of antigen-specific B cells into memory B cells and plasma cells. Produced HLA antibody (DSA) causes antibody-mediated rejection. Two approaches to predicting DSA production are shown in the process of allorecognition.
(1) T cell receptor of CD4+ T cell recognizes HLA molecules (T cell epitopes) presented by recipient APC, which is indicated by dotted circle. Magnitude of such indirect recognition of CD4+ T cells can be evaluated by recently-developed *in silico* prediction of indirectly recognizable HLA-derived peptides, the so-called PIRCHE algorithm.
(2) B cell receptor recognizes amino acid residues (eplet, B cell epitopes) on the molecular surface of donor HLA antigens, which is indicated by solid circle. The degree of epitope mismatch is evaluated by HLAMatchmaker which defines antibody-accessible amino acid polymorphism.

APC, antigen presenting cell; TCR, T cell receptor; BCR, B cell receptor; DSA, donor specific antibody; PIRCHE, predicted indirectly recognizable HLA epitopes.
