# Supplementary material for: Analysis of T and B Cell Epitopes to Predict the Risk of de novo Donor-Specific Antibody (DSA) Production After Kidney Transplantation: A Two-Center Retrospective Cohort Study
Source: Front Immunol. 2020 Aug 27;11:2000. doi: 10.3389/fimmu.2020.02000 (PMC7481442; doi:10.3389/fimmu.2020.02000)
Supplement: Supplementary file 2 [file Image_2.pdf]

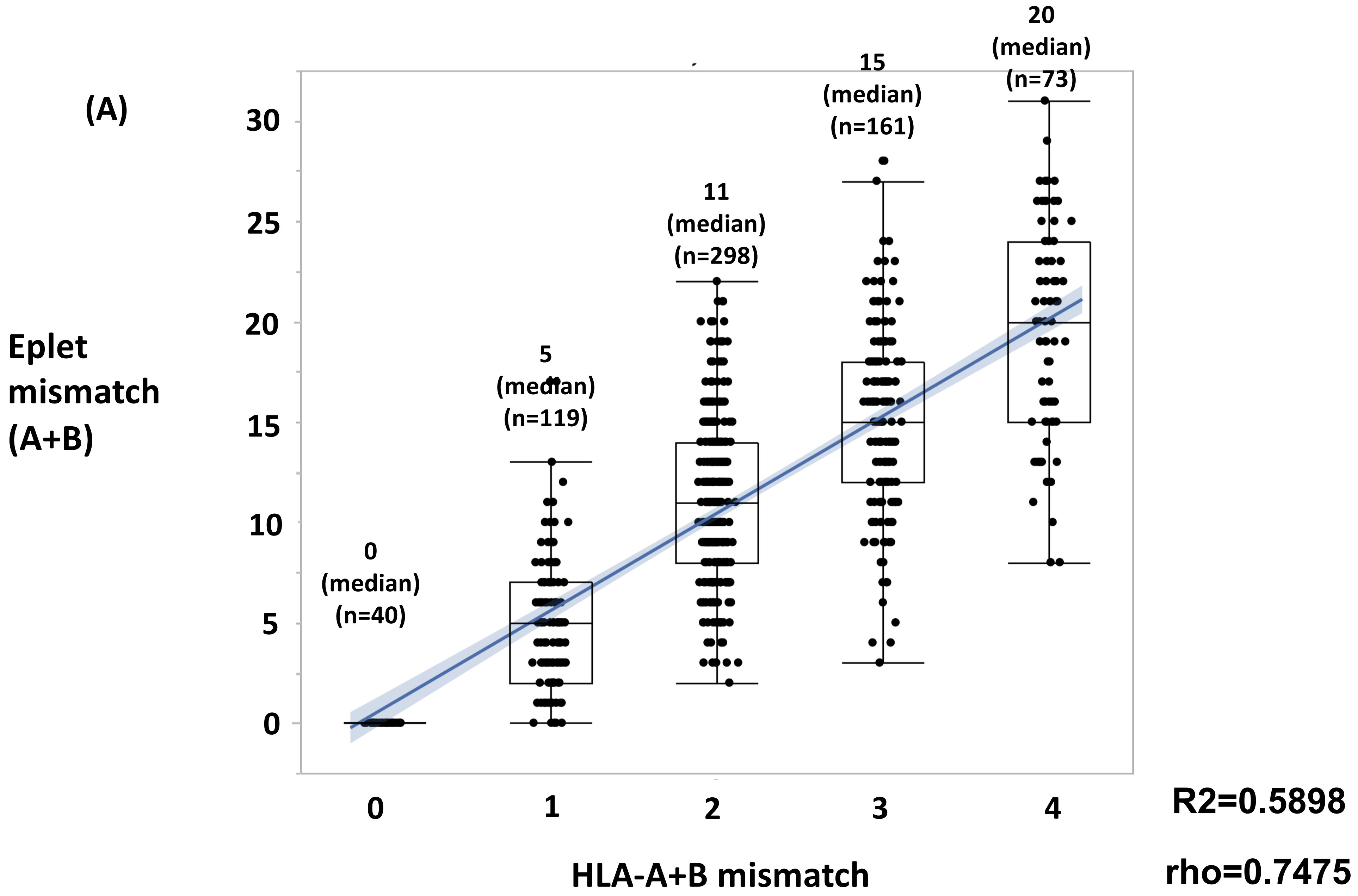

Supplementary Figure 2A. Association between classical HLA-A, B mismatch and eplet mismatch (A+B)

Box plots show minimum, first quartile, median, third quartile and maximum. Outliers are also displayed.
