# Supplementary material for: Analysis of T and B Cell Epitopes to Predict the Risk of de novo Donor-Specific Antibody (DSA) Production After Kidney Transplantation: A Two-Center Retrospective Cohort Study
Source: Front Immunol. 2020 Aug 27;11:2000. doi: 10.3389/fimmu.2020.02000 (PMC7481442; doi:10.3389/fimmu.2020.02000)
Supplement: Supplementary file 5 [file Image_5.pdf]

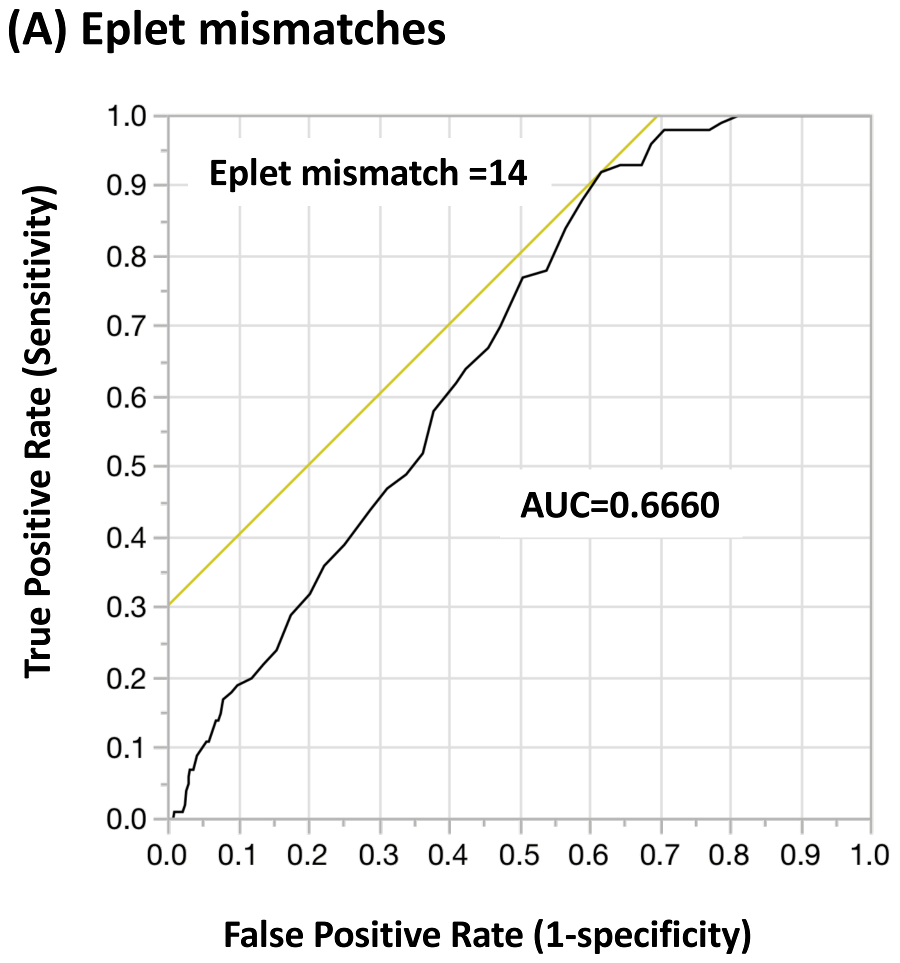

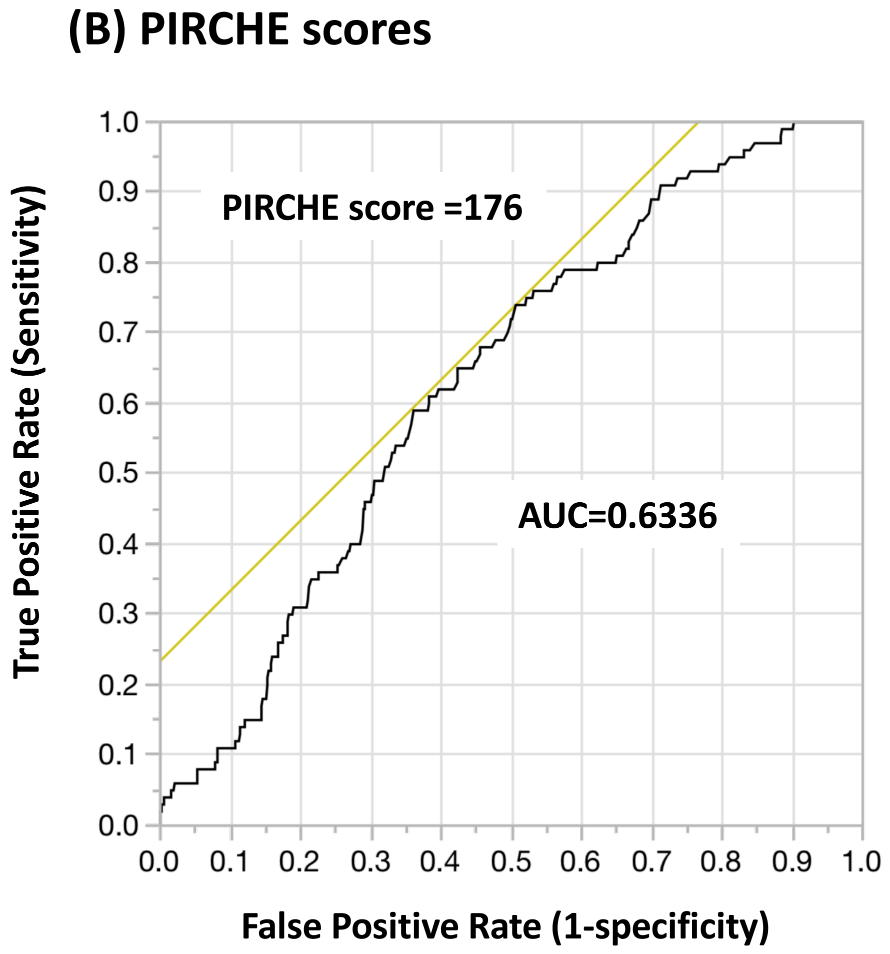

Supplementary Figure 3. ROC curves for prediction of de novo DSA production

ROC (receiver operating characteristic) curve and AUC (area under the curve) are by (A) eplet mismatches and (B) PIRCHE scores.
